# Novel Strain of the Chronic Wasting Disease Agent Isolated From Experimentally Inoculated Elk With LL132 Prion Protein

**DOI:** 10.1038/s41598-020-59819-1

**Published:** 2020-02-21

**Authors:** Jo Moore, Trudy Tatum, Soyoun Hwang, Catherine Vrentas, M. Heather West Greenlee, Qingzhong Kong, Eric Nicholson, Justin Greenlee

**Affiliations:** 1 USDA, Agricultural Research Service, National Animal Disease Center, Virus and Prion Research Unit, Ames, 50010 USA; 2 Iowa State University, Department of Biomedical Sciences, Ames, 50010 USA; 3 Case Western Reserve University, Departments of Pathology and Neurology, Cleveland, 44106 USA

**Keywords:** Neurodegeneration, Prion diseases

## Abstract

Chronic wasting disease (CWD) is a fatal, progressive disease that affects cervid species, including Rocky mountain elk (*Cervus elaphus nelsoni*). There are 2 allelic variants in the elk prion protein gene: L132 (leucine) and M132 (methionine). Following experimental oral challenge with the CWD agent incubation periods are longest in LL132 elk, intermediate in ML132 elk, and shortest in MM132 elk. In order to ascertain whether such CWD-infected elk carry distinct prion strains, groups of *Tg12* mice that express M132 elk prion protein were inoculated intracranially with brain homogenate from individual CWD-infected elk of various genotypes (LL132, LM132, or MM132). Brain samples were examined for microscopic changes and assessment of the biochemical properties of disease-associated prion protein (PrP^Sc^). On first passage, mice challenged with LL132 elk inoculum had prolonged incubation periods and greater PrP^Sc^ fibril stability compared to mice challenged with MM132 or LM132 inoculum. On second passage, relative incubation periods, western blot profiles, and neuropathology were maintained. These results suggest that the CWD prion isolated from LL132 elk is a novel CWD strain and that M132 PrP^C^ is able to propagate some biophysical properties of the L132 PrP^Sc^ conformation.

## Introduction

Chronic wasting disease (CWD) is a fatal, progressive, neurodegenerative disease that has been reported in a number of cervid species including Rocky Mountain elk (*Cervus elaphus nelsoni*) and white-tailed deer (*Odocoileus virginianus*). Chronic wasting disease belongs to a group of diseases known as the transmissible spongiform encephalopathies (TSEs), or prion diseases, that are a group of neurodegenerative diseases in which a key feature is the accumulation of disease-associated prion protein (PrP^Sc^) in the the brain.

The prion protein gene (*PRNP*) of Rocky Mountain elk encodes for either methionine (M) or leucine (L) at codon 132^1,2^. The amino acid expressed at codon 132 greatly affects incubation periods in elk after experimental oral inoculation with the chronic wasting disease agent: elk expressing prion protein homozygous for leucine at codon 132 (hereafter referred to as LL132 elk) incubate approximately 1.5 times longer than LM132 elk and 3 times longer than MM132 elk^3,4^. We recently demonstrated that these variations in incubation period may be influenced by genotype-associated differences in the relative amount of PrP^Sc^ in the brain and biochemical properties of PrP^Sc^, including fibril stability and amyloid formation rate^5^.

TSE strains may be differentiated by host range, incubation period, clinical presentation, patterns of immunoreactivity or microscopic lesions in the brain, or the physiochemical properties of the PrP^Sc^ itself (reviewed in^6^). Prions lack genomic DNA to propagate strain information, and the strain properties are believed to be enciphered by the structure of the PrP^Sc^ conformer^7–11^. Two different strains of CWD have been identified when CWD from white-tailed deer was passaged into Syrian hamsters^12^, ferrets^13^, and transgenic mice expressing cervid PrP^14,15^. Natural CWD isolates from elk may contain either one^12,16,17^ or two^14^ strains.

To further explore the diversity of CWD strains in elk of different genotypes, brain homogenates from experimentally CWD-infected elk of the MM132, LM132 and LL132 genotypes were inoculated into transgenic mice expressing M132 prion protein^18^. Using this mouse model, we demonstrate that biological properties and biochemical characteristics of PrP^Sc^ in mice inoculated with brain homogenate from MM132 and LL132 elk are distinct from each other and similar to that in homologous donor elk. These differences are maintained on second passage suggesting that CWD-infected 132LL elk propagate a novel CWD strain that is distinct from the classic CWD strain of 132MM and 132 ML elk.

## Results

To investigate the properties of PrP^Sc^ from experimentally infected elk of different *PRNP* genotypes, brain material from CWD-infected elk was bioassayed in *Tg12* mice that express the M132 elk prion protein. When presenting bioassay results, the PrP^Sc^ label applied to mice (e.g. MM, LM, LL) refers to the prion protein genotype of the original donor elk, not the mouse itself.

### Incubation periods

**Figure 1:**
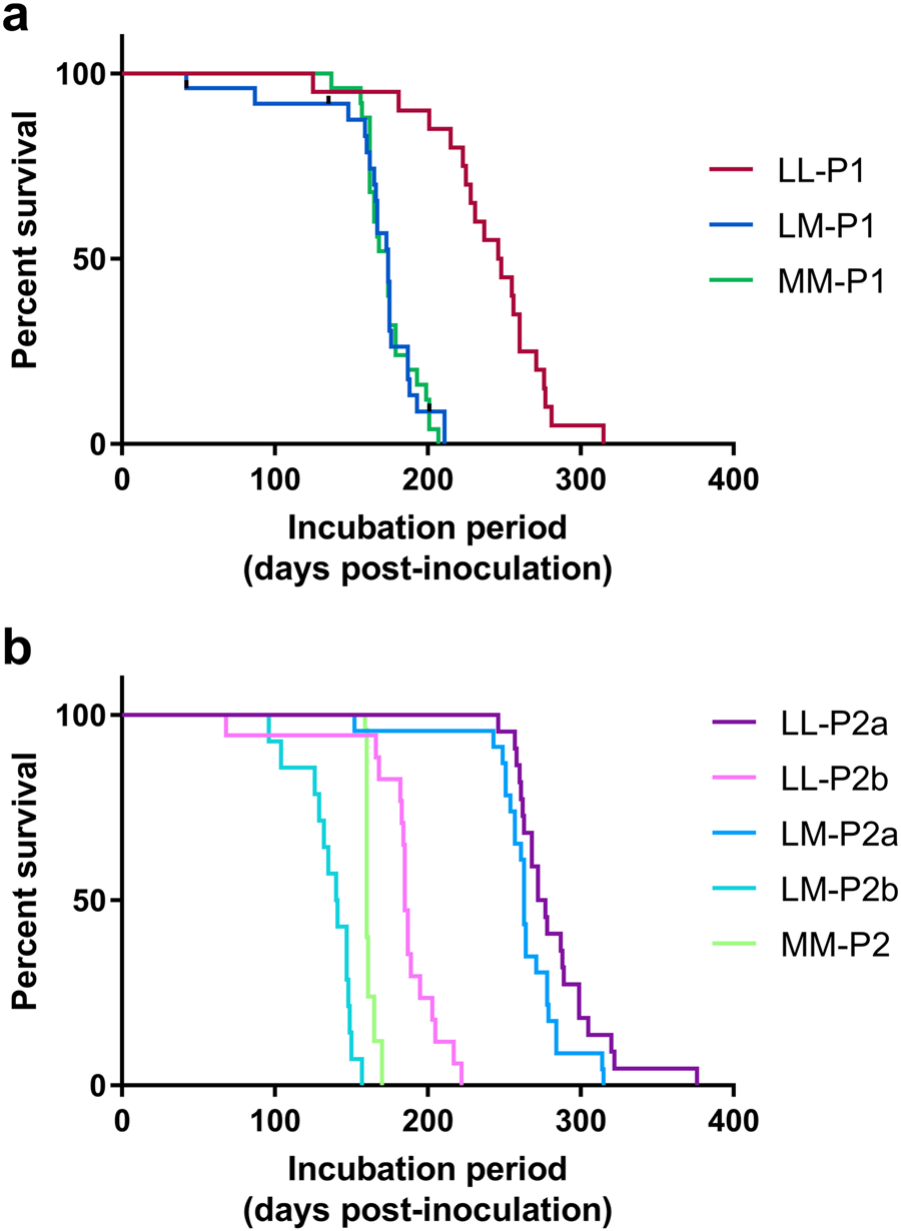
Survival curves for first (**a**) and second (**b**) passage of the agent of chronic wasting disease from elk of different prion protein genotypes. All transgenic mice express M132 elk prion protein. In mouse group names LL/LM/MM indicates the prion protein genotype of the elk donor for the first passage study i.e. LL132, LM132 and MM132 respectively.

On first passage, mean incubation periods for MM-PrP^Sc^ (from MM132 elk) and LM-PrP^Sc^ (from LM132 elk) in Tg12 mice (referred to as MM-P1 mice or LM-P1 mice) were 174 and 164 days post-inoculation (dpi), respectively. The mean incubation time for LL-PrP^Sc^ (from LL132 elk) in Tg12 mice (termed LL-P1) was significantly longer (241 dpi, Log-rank test *p* < 0.0001) (Table 1, Fig. 1). On second passage, mean incubation periods (263 dpi) for LM-PrP^Sc^ in Tg12 mice (LM-P2a mice) were 20 days shorter than LL-PrP^Sc^ in Tg12 mice (LL-P2a mice; 283 dpi, Log-rank test *p* = 0.0387) and 101 days longer than MM-PrP^Sc^ (MM-P2 mice; 162 dpi, Log-rank test *p* < 0.0001) (Table 1, Fig. 1).

**Table 1:** Transmission of elk CWD prions to mice expressing M132 elk prion protein.

PrP^Sc^ donor genotype	Mouse group	Passage	Mouse donor EIA OD	Attack rate (inoculated/EIA positive)	Incubation period (mean days ± SD)
MM132	MM-P1	First	n/a	25/26	174 ± 3.4^b^
	MM-P2	Second	4.0	25/25	162 ± 0.7^c^
LM132	LM-P1	First	n/a	21/24	164 ± 7.4^b^
	LM-P2a	Second	1.8	23/23	263 ± 6.2^a^
	LM-P2b	Second	4.0	14/14	136 ± 4.5^d^
LL132	LL-P1	First	n/a	13/13	241 ± 8.9^a^
	LL-P2a	Second	0.6	23/25	283 ± 6.1^e^
	LL-P2b	Second	4.0	17/18	183 ± 7.8^f^

M, methionine; L, leucine; EIA OD, enzyme immunoassay optical density result; SD, standard deviation; n/a, not applicable; Incubation periods, statistically significant differences are indicated by different superscript letters.

The results of the LM-P2a bioassay were unexpected since generally incubation periods for second passage are similar to or shorter than first passage. On revisiting the first passage data, it was discovered that the mouse from the LM-P1 group that had been used as a donor for the LM-P2a study had a low EIA optical density (OD) result (OD = 1.8). The low EIA OD result indicates a relatively lower amount of PrP^Sc^ in the brain that we hypothesized could be the cause of the unexpectedly longer mean incubation period for the LM-P2a group. Therefore, we determined the amount of PrP^Sc^ in the brain homogenate from each donor mouse using EIA (Table 1). For studies that the donor mouse of the original passage had a low EIA OD (LL-P2a and LM-P2a), additional studies were performed using donors with OD = 4.0 (LL-P2b and LM-P2b) that is the highest measurable value in this test. In both additional studies the mean incubation periods were shorter than the first passage studies (Log-rank test, both p < 0.0001) (Table 1). Therefore, the LM-P2b and LL-P2b data are more comparable to that of MM-P2.

### Amount of PrP^Sc^ versus incubation period in Tg12 mice

We observed a correlation between group mean incubation period and the amount of PrP^Sc^ present in the donor inoculum. Higher amounts of PrP^Sc^ in the inoculum was associated with shorter incubation periods and vice versa (Fig. S1). The relative amount of PrP^Sc^ in elk brain homogenate used to inoculate mice in the first passage studies was approximately 10 fold higher (Fig. S1a) than that from *Tg12* mouse donors used to inoculate mice in the second passage studies (Fig. S1b).

### Western blotting

**Figure 2:**
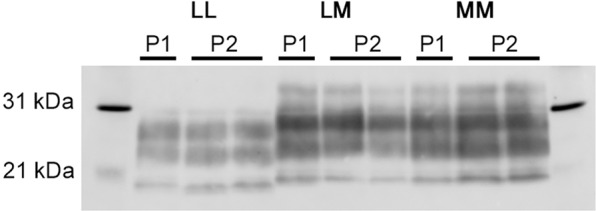
Western blot analysis of brain homogenates from mice challenged with brain homogenate from elk with chronic wasting disease. Lanes 1 and 11, markers. All mice express M132 elk prion protein. LL/LM/MM indicates the prion protein genotype of the elk donor for the first passage study i.e. LL132, LM132 and MM132 respectively. P1, first passage (elk to mouse); P2, second passage (mouse to mouse). Anti-prion protein antibody 6H4.

Western blot migration patterns were consistent across first and second passages of each elk inoculum (Fig. 2). No differences in western blot migration pattern were observed between LL-P2a and LL-P2b groups, or LM-P2a and LM-P2b groups (data not shown). PrP^Sc^ migration patterns for LM and MM mice were similar with the lower (unglycosylated) band migrating at approximately 20 kDa. PrP^Sc^ from LL mice ran somewhat lower, with the lower band at approximately 18 kDa. The relative positions of the lower bands in LL and MM/LM mice are consistent with the differences in banding pattern observed between LL132 and MM/LM132 elk^3^.

### Histopathology

To investigate whether the inoculum source influenced brain pathology, we scored the severity of spongiform change in defined grey and white matter neuroanatomical areas for each mouse and calculated mean vacuolation scores for each group of mice to create lesion profiles.

Lesion profiles for all groups were broadly similar for all inoculation groups, although some minor differences were observed (data not shown). Amongst first passage groups, the severity of vacuolation in the neocortex at the level of the thalamus was somewhat lower in mice inoculated with LL132 elk brain homogenate. Amongst second passage groups, the overall severity of vacuolation was greatest in MM-P2 mice, particularly in the medulla, cerebellum, hippocampus and interpeduncular nucleus. Compared to other second passage groups, LM-P2a mice showed less severe vacuolation in the neocortex at the level of the thalamus and caudate putamen.

**Figure 3:**
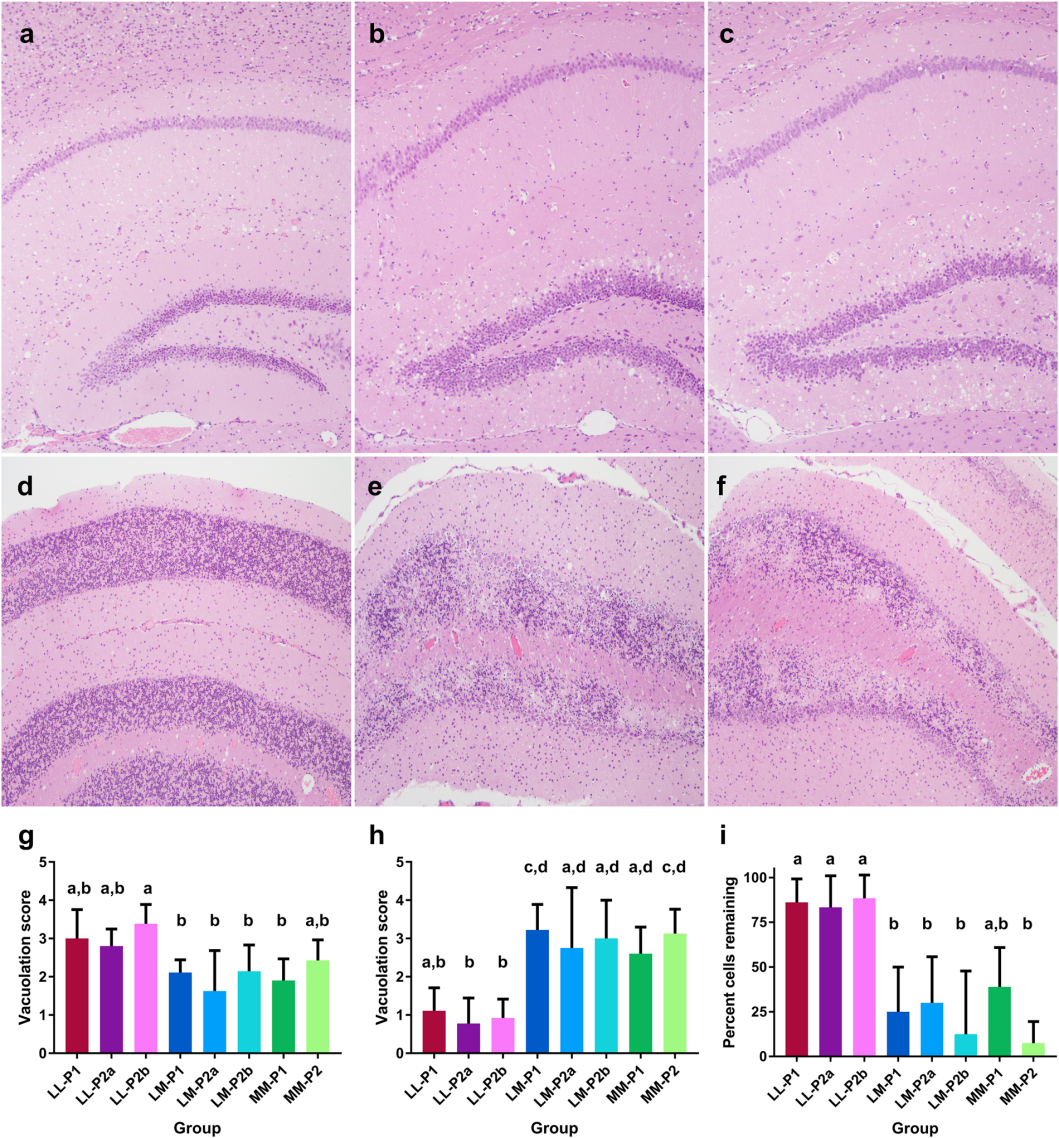
Microscopic changes in the hippocampus (**a–c**, **g,h**) and cerebellum (**d–f**, **i**) of cervidized mice inoculated with brain material from LL132 (**a,d**), LM132 (**b,e**) or MM132 (**c,f**) CWD-affected elk. Graphs depict the severity of vacuolation in the CA1 area (**g**) and dentate gyrus (**h**) of the hippocampus, and magnitude of reduction of granule cells in the cerebellum (**i**). Error bars reflect the SEM (standard error of the mean). Statistically significant differences are indicated by column labels that do not share the same letters. All mice express M132 elk prion protein; LL/LM/MM indicates prion protein genotype of the elk donors for the first passage study i.e. LL132, LM132 and MM132 respectively; P1, first passage (elk to mouse); P2, second passage (mouse to mouse). All photomicrographs original magnification 10×.

Although the overall vacuolation scores for the hippocampus were similar for all inoculation groups, differences were observed in the distribution of vacuolation within the hippocampus (Fig. 3a–c). To investigate this further, the severity of vacuolation in 3 hippocampal regions (dentate gyrus, CA3 and CA1) was scored using the same 0–5 scale as for vacuolation lesion profiling in other brain areas. In LL-PrP^Sc^ mice, vacuolation was more severe in the CA1 area and less severe in the dentate gyrus (Fig. 3a,g). This pattern was reversed in LM-PrP^Sc^ and MM-PrP^Sc^ mice such that vacuolation was more severe in the dentate gyrus and less severe in the CA1 area (Fig. 3b,c,h). Subjectively, the severity of vacuolation in the CA1 area was higher in LL-PrP^Sc^ mice as compared to LM-PrP^Sc^ and MM-PrP^Sc^ mice. However, these differences were only statistically significant for the LL-P2b group (ANOVA Kruskal-Wallis, p < 0.04). The severity of vacuolation in the CA3 area was similar across all groups (ANOVA Kruskal-Wallis, all p > 0.8). These results were maintained across first and second passage.

While performing vacuolation lesion profiling, neuronal loss in the granular layer of the cerebellar cortex was noted in some animals. To investigate this further, the severity of granule cell reduction was scored on a scale of 0–4 to reflect the surface area affected: 0, normal cellularity; score 1, 2, 3, 4, reduced cellularity in 1–25%, 26–50%, 51–75% and 76–100% of the section, respectively. Loss of granule cells was absent to mild (mean score = 0.6) in LL-PrP^Sc^ mice (Fig. 3d,i) and moderate to severe in LM-PrP^Sc^ (mean score = 3.4) and MM-PrP^Sc^ (mean score = 2.9) mice (Fig. 3e,f and i). Loss of granule cells was less severe in MM-P1 mice (mean score = 2.0) compared to MM-P2 mice (mean score = 3.7) (Fig. 3i). The Purkinje cells and cells in the molecular layer were normal.

### Immunohistopathology

**Figure 4:**
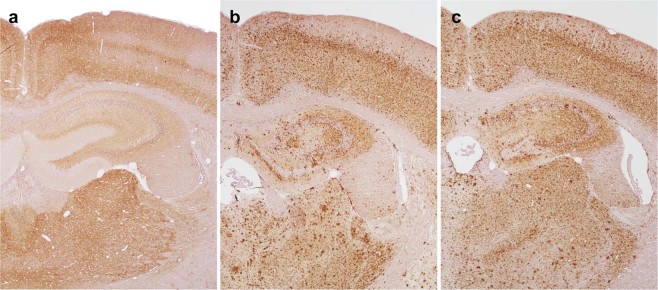
Patterns of PrP^Sc^ immunoreactivity in brains from mice from the second passage of CWD-affected elk brain homogenate from LL132 (**a**), LM132 (**b**) and MM132 (**c**) elk. In LL mice (**a**) immunoreactivity is present as diffuse immunolabeling of the neuropil while in LM (**b**) and MM (**c**) mice there are widespread medium to large aggregates in addition to diffuse immunolabeling of the neuropil. PrP^Sc^ detected with mAb 6H4.

PrP^Sc^ immunoreactivity was present throughout the brain of all mice examined. The pattern of PrP^Sc^ deposition in the brain was similar for MM-PrP^Sc^ (MM-P1, MM-P2) and LM-PrP^Sc^ (LM-P1, LM-P2a, LM-P2b) mice and different for LL-PrP^Sc^ (LL-P1, LL-P2a, LL-P2b) mice (Fig. 4) and these patterns were consistent across first and second passage.

In LL-PrP^Sc^ mice (Fig. 4a), immunoreactivity was present as diffuse immunolabeling of the neuropil with scattered aggregates in the molecular layer, and occasionally the granular cell layer, of the cerebellum. In the hippocampus, immunoreactivity was more severe in the CA1 and CA3 areas and less severe in the dentate gyrus.

In MM-PrP^Sc^ (Fig. 4b) and LM-PrP^Sc^ (Fig. 4c) mice, the PrP^Sc^ deposition pattern was characterized by widespread medium to large aggregates and diffuse immunolabeling of the neuropil. In the cerebellum, immunoreactivity was intense in the granular cell layer and mild to moderate in the molecular layer. In the hippocampus, immunoreactivity was moderate to severe in the dentate gyrus and CA3 area, and mild to moderate in the CA1 area. Moderate to intense immunoreactivity was present in the ependymal cells of the mesencephalic aqueduct and third ventricle.

### Fibril stability of PrP^Sc^ from *Tg12* mice

**Figure 5:**
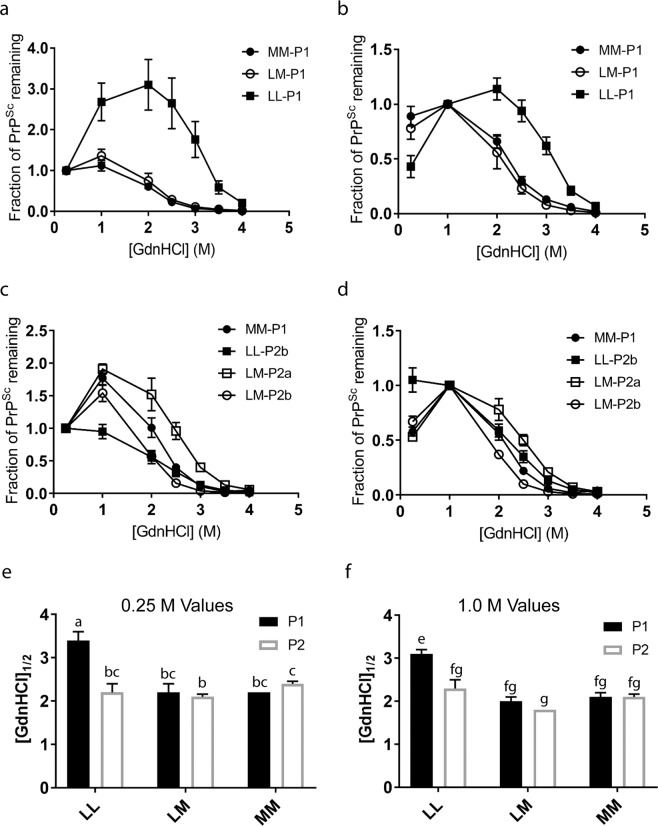
Fibril stability of PrP^Sc^ from transgenic mice expressing elk M132 prion protein. (**a,b**) Stability of PrP^Sc^ from first passage mice, normalized to either the 0.25 M data point (**a**) or the 1 M data point (**b**). Curves are averaged across 4 (MM, LL groups) or 5 (LM group) mice per group with the curve for each mouse reflecting the mean across technical replicates. (**c,d**) Stability of PrP^Sc^ from second passage mice, normalized to either the 0.25 M data point (**c**) or the 1 M data point (**d**). Curves are averaged across 5 (LM-P2a, MM-P2 groups), 6 (LM-P2b), or 8 (LL-P2b) mice, with the curve for each mouse reflecting the mean across technical replicates. [GdnHCl]_1/2_ = Fraction of PrP^Sc^ remaining (0.5). In each case, error bars reflect the SEM (standard error of the mean). (**e,f**) Comparison of [GdnHCl]_1/2_ values of PrP^Sc^ across first and second passage mice, normalized to either the 0.25 M data point (**e**) or the 1 M data point (**f**), as displayed in Table 2. LM values represent those collected from LM-P2b mice. Letters were added to correspond to the assessment of statistical significance described in Table 2.

The fibril stability of PrP^Sc^ in homogenized brain from selected mice from each inoculum x passage combination was measured using an EIA-based stability assay. This assay is based on the use of the IDEXX HerdChek EIA platform to recognize misfolded protein; as a result, PK digestion is not involved, and the assay can detect both PK-sensitive and PK-resistant PrP^Sc^ populations (therefore retaining a broader pool of PrP^Sc^ for analysis). For samples from the first passage of elk brain into the mice, the LL-P1 mice exhibited a large increase in signal between 0.25 and 1 M GdnHCl conditions, and a relatively low absolute signal at the 0.25 M GdnHCl condition. This likely reflects increased binding of PrP^Sc^ to the capture surface or increased epitope exposure over the GdnHCl range in question, either of which would result an increased signal in the assay. It is worth noting that this may also influence determination of EIA signal in addition to differences in total PrP^Sc^ concentration in brain tissue. We address this in the determination of fibril stability by analyzing the data in two ways: using normalization of the EIA signal to the 0.25 M point (Fig. 5a,c), and using normalization to the 1 M point (Fig. 5b,d).

On first passage, the curves (Fig. 5a,b) and [GdnHCl]_1/2_ values (Table 2) were similar for the MM-P1 and the LM-P1 mice, and distinct for the LL-P1 mice. [GdnHCl]_1/2_ values were significantly different between the MM-PrP^Sc^ and LL-PrP^Sc^ mice (p = 0.0007 in an unpaired t-test assuming unequal variances) (Fig. 5e,f). Therefore, the relative stability of PrP^Sc^ from MM132 versus LL132 elk brain was propagated on first passage in transgenic M132 elk mice.

**Table 2:** Summary of [GdnHCl]_1/2_ values for each mouse group.

Mouse Group	[GdnHCl]_1/2_ values	
	Normalized to 0.25 M	Normalized to 1 M
LL-P1	3.4 ± 0.2^a^	3.1 ± 0.1^e^
LM-P1	2.2 ± 0.2^bc^	2.0 ± 0.2^fg^
MM-P1	2.2 ± 0.04^bc^	2.1 ± 0.1^fg^
LL-P2b	2.1 ± 0.2^bc^	2.2 ± 0.1^fg^
LM-P2a	2.6 ± 0.1^c^	2.5 ± 0.1^f^
LM-P2b	2.1 ± 0.06^b^	1.8 ± 0.04^g^
MM-P2	2.4 ± 0.06^c^	2.1 ± 0.07^fg^

[GdnHCl]_1/2_ is defined as the concentration of GdnHCl at which the PrP^Sc^ signal was reduced by half of the signal at the normalization concentration (0.25 M or 1 M GdnHCl). [GdnHCl]_1/2_ values were determined for each individual mouse and numbers presented in the table reflect mean values for all mice tested in each group + /− the standard error of the mean. For groups with shared superscripts within a column, differences between the means (as assessed by the Games-Howell post-hoc test) were not significant (P < 0.05). Note that since 0.25 M and 1 M values were analyzed separately, different superscripts between the two columns do not indicate assessment of differences across columns.

Next, we examined the fibril stability of PrP^Sc^ in the second passage mouse brains, using analysis with 0.25 M (Fig. 5c) and 1 M (Fig. 5d) normalization. Due to the relatively low starting signals in the LL-P2a brains, only the LL-P2b samples were used to assess LL-P2 stability. The resulting [GdnHCl]_1/2_ values (Table 2) demonstrate that upon the second passage into transgenic M132 mice, the fibril stability profiles of the mice converge (Fig. 5c–f). This suggests that although the PK cleavage properties of PrP^Sc^ are maintained across first and second passage (Fig. 2), the fibril stability of PrP^Sc^ undergoes conformational changes on second passage resulting in convergence of stability curves across all experimental groups, despite the retention of the western blotting pattern. The LM-P2b fibril stability was lower than observed for the MM-P2 group that could correlate with the faster incubation time observed for this group.

### Real time quaking-induced conversion

To determine if RT-QuIC can be used to detect PrP^Sc^ in CWD infected mouse brains and to investigate potential differences in prion seeding activity, RT-QuIC reactions using mature length recombinant 132 L or 132 M elk prion protein substrates were seeded with CWD infected and non-infected mouse brain homogenates.

**Figure 6:**
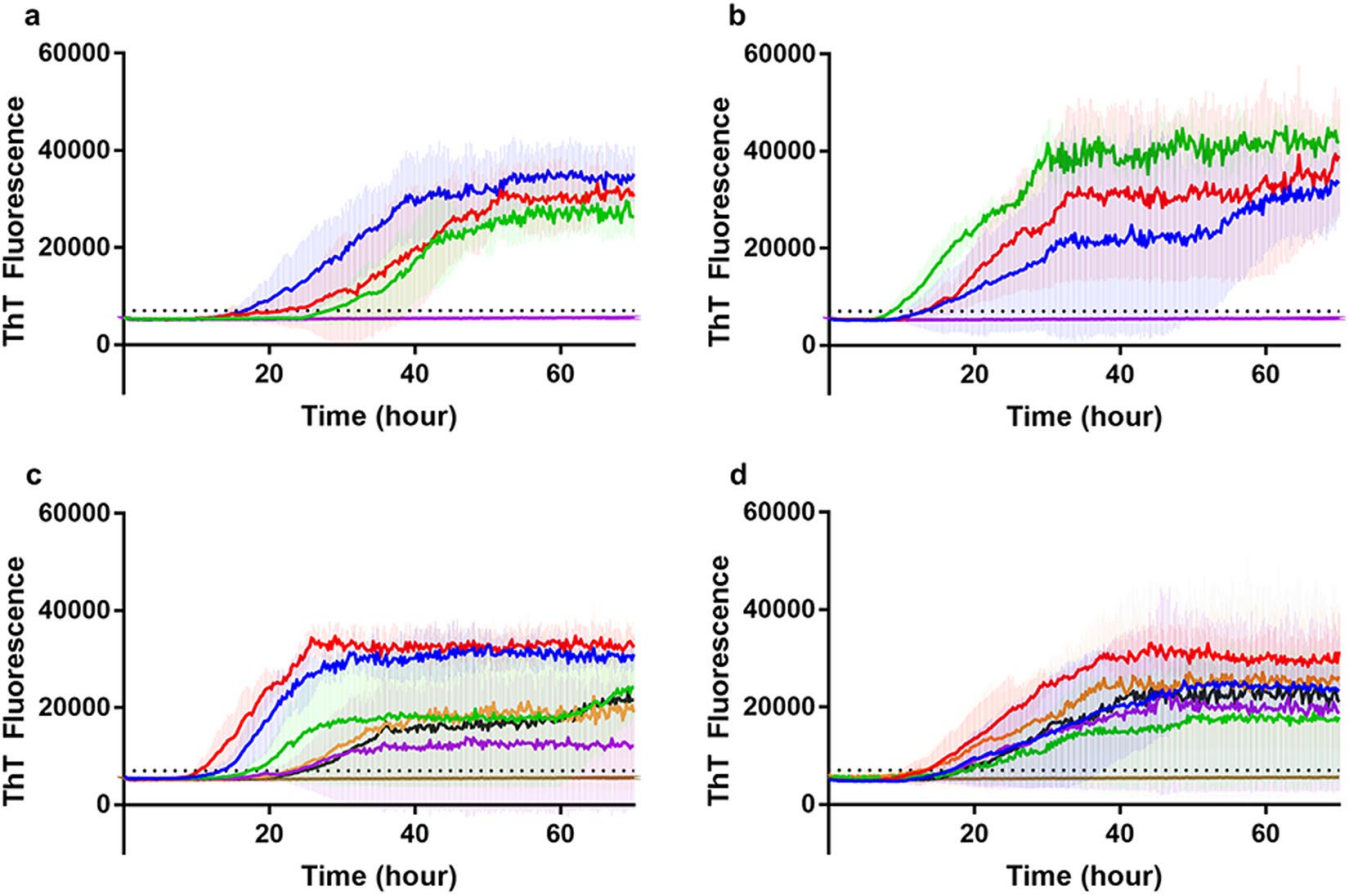
RT-QuIC detection of seeding activity in brain samples from transgenic mice expressing M132 elk prion protein, first passage (**a,b**) and second passage (**c,d**). Substrate is full-length recombinant elk prion protein 132 L (**a,c**) or 132 M (**b,d**). Dashed horizontal line indicates the positive threshold above which a sample is considered positive. (**a,b**) Mice were inoculated with brain homogenate from CWD-affected elk of different genotypes: MM132 (green), LM132 (red), LL132 (blue); negative seed (purple). (**c,d**) Mice were inoculated with brain homogenate from first passage mice that were inoculated with brain homogenate from CWD-affected elk of different genotypes: MM132 (yellow, black), LM132 (green, purple), LL132 (blue, red); negative seed (tan). Each curve represents the mean ThT fluorescence of eight technical replicates for one mouse. RT-QuIC reaction mixtures were seeded with 10^−3^ dilutions of normalized brain homogenate. A final concentration of 300 mM NaCl was used with each substrate. The positive threshold was calculated as ~7,000 relative fluorescence units of brain homogenate from normal mice.

Seed prepared from brains from first passage mice showed an increase in ThT fluorescence in both substrates (132 L or 132 M). Normal brain homogenates did not result in an increase in ThT fluorescence. Using 132 L substrate (Fig. 6a), the lag time for reactions seeded with LL-P1 mouse brain (18 hours) was shorter than that for LM-P1 (24 hours) and MM-P1 (30 hours) samples. Using 132 M substrate (Fig. 6b), assays seeded with MM-P1 mouse brain homogenate produced the shortest lag time (10 hours) while lag times for LM-P1 and LL-P1 samples were longer (both 15 hours).

When RT-QuIC reactions were seeded with brain homogenate from second passage mice, samples from all mice showed an increase in ThT fluorescence in both substrates (132 L or 132 M). Using 132 L substrate (Fig. 6c), LL-P2a samples resulted in shorter lag times (12 and 15 hour) and higher fibril formation compared to LM-P2a (20 and 24 hours) and MM-P2 samples (25 and 27 hours). Using the 132 M substrate (Fig. 6d), the lag times for all mice were similar.

## Discussion

In this study, we demonstrate that intracranial inoculation of transgenic mice expressing M132 elk prion protein (*Tg12*) with brain homogenate from elk of different *PRNP* genotypes that were experimentally infected with CWD results in clinical disease and accumulation of misfolded prion protein (PrP^Sc^). Compared to mice inoculated with MM132 elk brain homogenate, LL132 homogenate results in disease with a longer incubation time, lower migration pattern on western blot, more stable PrP^Sc^ fibrils as determined by an EIA-based stability assay, and fewer PrP^Sc^ aggregates in the brain as observed by immunohistochemistry (IHC). The disease phenotype in mice inoculated with LM132 elk brain homogenate is largely similar to that in mice inoculated with MM132 elk brain homogenate. Therefore, the phenotype of disease in mice appears to be strongly influenced by the *PRNP* genotype of the elk donor, although other factors, for example the amount of PrP^Sc^ in the donor inoculum, also play a role.

We have previously reported *PRNP* genotype-driven differences in disease expression in elk experimentally inoculated with the agent of CWD^3–5^. Compared to MM132 elk, disease in LL132 elk is characterized by prolonged incubation periods, lower migration pattern on western blot, higher fibril stability, and differences in the pattern of PrP^Sc^ accumulation in the brain^3,5^. With the exception of fibril stability, these phenotypic features were maintained when brain homogenate from CWD-infected elk was bioassayed in *Tg12* mice, suggesting that the LL132 isolate has novel CWD strain characteristics.

All three donor elk for the first passage mouse studies were inoculated with brain homogenate prepared from pooled brain material from one MM132 and one LM132 elk (equal parts MM132 and LM132 donor tissue)^5^ . Therefore it is possible that a mixture of prion strains was propagated in the donor elk. Although the sample size was small, the disease phenotype was consistent across multiple animals of the same *PRNP* genotype which makes the de novo emergence of a prion strain, as described previously for white-tailed deer, unlikely^15^. End-point titration of the all three elk donor inocula in *Tg12* mice could help to tease out individual strains from a mixture.

Similar patterns of PrP^Sc^ immunoreactivity and microscopic lesions were observed in the brains of *Tg12* mice inoculated with brain homogenate from CWD-infected elk of the MM132 or LM132 genotypes (hereafter, referred to as MM-PrP^Sc^ and LM-PrP^Sc^ mice, respectively), while a different pattern was observed in the brains of mice inoculated with LL132 elk brain homogenate (LL-PrP^Sc^ mice). These results are consistent with genotype-associated differences in pathology seen in donor elk^5^. For example, large aggregates and plaque-like PrP^Sc^ accumulations were observed throughout the brain of MM-PrP^Sc^ and LM-PrP^Sc^ mice, while immunoreactivity in LL-PrP^Sc^ mice was mostly diffuse. In donor elk, PrP^Sc^ accumulation in the brains of MM132 and LM132 elk was primarily neuropil-associated, while in LL132 elk, there was prominent PrP^Sc^ immunoreactivity associated with neurons and astrocytes^4,5^. Therefore, although the morphologic types of PrP^Sc^ immunolabeling are different in mice and elk, the *PRNP* genotype-associated grouping of PrP^Sc^ deposition patterns – that is, similar patterns in MM-PrP^Sc^ and LM-PrP^Sc^ mice and a different pattern in LL-PrP^Sc^ mice – are consistent across both elk and *Tg12* mice.

Using standardized vacuolation lesion profiling techniques^19^, we were not able to detect any significant differences between mouse groups. However, in the hippocampus, spongiform change was more severe in the dentate gyrus in MM-PrP^Sc^ and LM-PrP^Sc^ mice, while in LL-PrP^Sc^ mice, the CA1 area was more affected. A second microscopic change was observed in the cerebellum, where there was a marked reduction in the number of granule cells in MM-PrP^Sc^ and LM-PrP^Sc^ mice, while this change was mild to absent in LL-PrP^Sc^ mice. These results indicate that prion isolates from elk of different genotypes target and cause pathology in different populations of cells in the brain.

In donor elk, similarities in disease expression in MM132 and LM132 elk are thought to be due to a high proportion of the PrP^Sc^ in LM132 elk being M132^5^. This hypothesis is extrapolated from studies in scrapie-affected *PRNP* heterozygous sheep demonstrating that although expression of the host cellular form of the prion protein (PrP^C^) is equal for each *PRNP* allele, conversion of PrP^C^ to the disease-associated form (PrP^Sc^) is more efficient for the PrP^C^ moiety that confers a greater susceptibility to disease^20,21^. Since *Tg12* mice express PrP^C-132M^, patterns of disease expression on first passage are likely driven by sequence similarities or differences between the PrP^Sc^ in the elk donor inoculum and the PrP^C^ in the *Tg12* mice^22^. At second passage, all donor mice are *Tg12*, so all donor PrP^Sc^ is M132. Therefore, patterns of disease expression on second passage are probably associated with sequence-independent differences in PrP^Sc^ conformation^7–11^.

The relationship between PrP^Sc^ fibril stability in guanidinium hydrochloride (GdnHCl) and incubation time varies between host species and prions strains^23–30^. In CWD-affected elk, longer incubation periods were associated with higher fibril stability and fibril stabilities of PrP^Sc^ from MM132 and LM132 elk were similar^5^. These relationships were maintained on first passage (P1) of elk brain homogenate in *Tg12* mice and indicate that the M132 elk prion protein (as expressed in mice) can adopt the higher stability PrP^Sc^ conformation. The relative fibril stabilities in our first passage mice are in contrast to those reported for first passage of elk CWD isolates in Tg(CerPrP-M132)1536^+/−^ mice, where fibrils from mice inoculated with LM132 elk brain homogenate were more stable and fibrils from mice inoculated with MM132 or LL132 were less stable and similar to each other^17^. Another point of difference is in the relationship between PrP^Sc^ fibril stability and the morphology of PrP^Sc^ accumulation in the brain. Previously, studies in mice using mouse-adapted prion strains reported an association between higher PrP^Sc^ fibril stabilities and aggregated and plaque-like PrP^Sc^ accumulations, while isolates with lower fibril stabilities produce diffuse PrP^Sc^ deposits^25,27^. These results are in contrast to our findings where higher fibril stability of PrP^Sc^ from mice inoculated with CWD prions from LL132 elk brain is associated with diffuse PrP^Sc^ accumulation. The discrepancy between these results and our findings could be due to prion isolate and/or recipient mouse line related factors.

On second passage (P2) there was a significant reduction in the fibril stability of PrP^Sc^ from LL-P2 mice as compared to LL-P1 mice such that the fibril stability was similar to MM-P2 mice. In spite of this, the western blot migration patterns for LL-P1 and LL-P2 mice were identical which indicates that the conformational changes leading to changes in fibril stability are independent of PK cleavage properties. This convergence in fibril stability properties is likely due to adaptation in the *Tg12* mouse model and/or prion strain selection.

For LL-P2a and MM-P2 mice the relationship between elk donor *PRNP* genotype and incubation period was similar across first and second passages. However, variability was observed between the LL-P2a and LL-P2b groups, and the LM-P2a and LM-P2b groups.

For the LL-P2a and LL-P2b groups, amyloid formation rates, western blot profiles, neuropathology and fibril stability results were similar. Therefore the prolonged mean incubation period of the LL-P2a group compared to the LL-P2b group can be attributed to a lower PrP^Sc^ titer in the LL-P2a inoculum as indicated by a low EIA optical density (OD) result.

The explanation for the differences observed between the LM-P2a and LM-P2b groups is less clear. Mice from both groups had similar amyloid formation rates, western blot profiles and neuropathology results and, consistent with the situation in the LL-P2 groups, the lower PrP^Sc^ titer of the LM-P2a inoculum likely contributed to the longer mean incubation periods observed in this group. However, the fibril stability of PrP^Sc^ from mice in the LM-P2a group was higher than both the LL-P2 and MM-P2 groups, while the fibril stability of PrP^Sc^ from mice in the LM-P2b group was lower.

When Tg(CerPrP-M132)1536^+/−^ mice were inoculated with brain homogenates from CWD-affected MM132 elk, different patterns of neuropathology were observed in mice that developed disease early in the incubation period (up to 250 days post-inoculation) as compared to mice that developed disease later (250–400 days post-inoculation), although all mice in the study had similar western blot migration patterns and glycoform ratios^14^. The incubation period of the donor mouse for the LM-P2a study was approximately 50% of the mean incubation period for the LM-P1 group, so perhaps the prion strain isolated from this mouse was a short incubation period CWD strain with different biological characteristics, in particular fibril stability, compared to long incubation period strain(s) present in mice that developed disease later. Furthermore, it is probable that the original LM132 elk brain homogenate contained a mix of PrP^Sc^ conformations (PrP^Sc-132M^ and PrP^Sc-132L^) that may have been differentially propagated on second passage. Unfortunately, the brain of the mouse selected for passage was not fixed for microscopic examination and the PrP^Sc^ titer was too low to perform the fibril stability assay, but further experiments are underway to investigate whether these apparent strain characteristics are maintained on subsequent passages.

We also utilized RT-QuIC to investigate the influence of donor elk genotype on PrP^Sc^ characteristics in first and second passage in transgenic mice expressing M132 elk prion protein. Using recombinant mature length elk prion protein (132 L and 132 M) as substrates, seed prepared from brains from mice inoculated with MM132 elk inoculum converted 132 M substrate more readily compared to LM132 or LL132 seeds. In contrast, the 132 L substrate was converted most efficiently by seed prepared from brains from mice inoculated with LL132 elk inoculum. Seed homogenates were normalized prior to testing so the observed differences in conversion efficiencies are not due to variation in the amount of PrP^Sc^ in the seed. These patterns of conversion efficiencies in first passage mice are similar to those observed in donor elk^5^.

On second passage, results using 132 L substrate were similar to first passage results. However, using 132 M substrate, lag times for PrP^Sc^ from mice from all challenge groups (MM-P2, LM-P2, LL-P2) were similar. Transgenic *Tg12* mice express M132 cellular prion protein (PrP^C^), so at second passage, both donor mouse PrP^Sc^ and recipient mouse PrP^C^ are homologous. This could be a possible explanation for the convergence of lag times for samples from all challenge groups when the 132 M substrate was used. However, the fact that elk donor genotype-associated differences in lag time are still observed when 132 L substrate is used, and that western blot profiles for LL-PrP^Sc^ mice are consistent across first and second passages and different from LM-PrP^Sc^ and MM-PrP^Sc^ mice, suggests that the M132 PrP^Sc^ in LL-P2 mice still maintains distinct biochemical characteristics compared to M132 PrP^Sc^ from LM-P2 and MM-P2 mice. Bioassay of elk brain homogenate in mice expressing L132 PrP^Sc^ would help to clarify the role of the L132 polymorphism in selection and emergence of prion strains in elk.

The current study lends evidence to the fact that there are multiple distinct strains of the CWD agent in elk. It cannot be entirely ruled out that the inoculum used in the original elk transmission study contained multiple strains. The inoculum used for the oral dosing was comprised of pooled material from two clinically ill elk: one elk of each the MM132 and ML132 genotypes. The infectous material came from a single premises and western blotting of the inoculum resulted in a single migration pattern. However, further analysis of the inoculum was not performed at the time of the original study and none of this inoculum is currently available for testing. Since results from the original transmission study in elk indicate that biochemical properties of of PrP^Sc^ from MM132 and LM132 elk are similar^5^, it seems likely that the second strain, if present in the original inoculum, was present as a minor component. Although two strains of the CWD agent have been previously described in naturally infected elk^14^, the lower western blot migration pattern of the LL132 strain described in the experimentally challenged elk reported here clearly distinguishes it from previously described strains. Thus it would appear that elk are able to amplify a total of at least three strains of CWD.

In previous studies of naturally occurring CWD in elk, one study^31^ reported similar western blot migration patterns for MM132 and LL132 elk and a different pattern for LM132 elk, while in another study no genotype-associated differences were observed between MM132, LM132 and LL132 elk^17^. Given this variation in western blot migration pattern in naturally infected elk it is not possible to be certain whether the lower migration pattern observed in our donor elk is an additional naturally occurring variant or represents an experimental epiphenomenon related to e.g. experimental inoculation with a high dose of pooled CWD infectivity. Bioassay of additional CWD isolates from naturally infected LL132 elk may help to improve our understanding of naturally occurring LL132 elk CWD strains.

To summarize, we have demonstrated that inoculation of mice expressing elk prion protein with brain homogenate from CWD-affected elk produces disease with different biological and biochemical characteristics depending on elk donor *PRNP* genotype. The phenotype of disease in mice inoculated with brain homogenate from MM132, LM132 and LL132 elk is similar to that observed in donor elk with regards to relative incubation period, PrP^Sc^ fibril stability, and western blot migration patterns. On second passage we observed a convergence in incubation time, fibril stability, and amyloid formation in 132 M substrate for MM and LL groups, presumably due to prion strain selection by the *Tg12* mouse model. However, differences in western blot profiles and neuropathology were maintained across first and second passages indicating that CWD from experimentally inoculated MM132 and LL132 elk represent distinct strains of CWD. Second passage of the LM132 isolate resulted in two distinct fibril stability profiles. These results provide further evidence of the existence of multiple strains of CWD in elk. If the novel LL132 strain that we have isolated from experimentally infected elk is recapitulated in naturally elk, the presence of this third strain of CWD would have the potential to complicate CWD management strategies in free-ranging and captive populations.

## Methods

### Ethics statement

This experiment was carried out in accordance with the Guide for the Care and Use of Laboratory Animals (Institute of Laboratory Animal Resources, National Academy of Sciences, Washington, DC). The protocol was approved by the Institutional Animal Care and Use Committee at the National Animal Disease Center (protocol numbers: ARS 2729 and ARS-2017-628).

### Inoculum preparation and animal procedures

Infectivity in brain tissue from selected elk was assayed via intracranial inoculation of Tg(ElkPrP-132M)Prnp^0/0^ mice (hereafter referred to as *Tg12* mice) that express M132 elk prion protein at approximately twofold the expression level of brain PrP in wild-type FVB mice^18^. CWD inoculum was prepared from the brains of experimentally challenged CWD-affected elk of the MM132, LM132, and LL132 genotypes. These elk had been challenged intracranially with pooled brain material from one MM132 and one LM132 elk, both of which had showed clinical signs of CWD^4^. The results of the elk challenge study have been reported previously^3–5^.

Mice were inoculated intracranially with 20 µL of 1% w/v brain homogenate from a single elk as described previously^32^. Mice were monitored daily and euthanized when they displayed unequivocal neurological signs (poor coordination, difficulty moving, unable to move, anorexia) or at the time of study termination (700 days post-inoculation). The incubation period was calculated as the number of days between inoculation and euthanasia.

Mouse brains were sectioned longitudinally, and 2/3 of the brain was fixed for microscopic examination and 1/3 was frozen at −80 C for biochemical assays. Tissue for microscopic examination were fixed in 10% buffered formalin, embedded in paraffin wax, sectioned at 5 μm, and stained with hematoxylin and eosin. Frozen tissue was tested for the presence of PrP^Sc^ using enzyme immunoassay and western blotting as described below.

For second passage, brain samples from mice were prepared as 10% w/v brain homogenates in phosphate buffered saline as described previously^33^.

### Histopathology

For each mouse, the severity of spongiform change was semi-quantified on a scale of 0–5 or 0–3 for 9 gray matter areas and 3 white matter areas, respectively, as described previously^19^. Lesion profiles were obtained by two observers scoring 7–13 positive (by enzyme immunoassay, see below) mice per inoculation group.

### Immunohistochemistry

For each mouse paraffin-embedded brain sections were immunostained manually for detection of PrP^Sc^ using the mouse anti-PrP monoclonal antibody 6C2 (CVI-WUR, Lelystad, Netherlands) as described previously^34^ with the following modifications: for antigen retrieval, rehydrated sections were incubated with 98% formic acid for 15 minutes and then autoclaved for 30 minutes in an antigen retrieval solution (DAKO Target Retrieval Solution, Dako Corp., Carpinteria, CA). Tissues were then blocked for 20 minutes at room temperature (Background Buster; Innovex, Lincoln, RI).

### Western blotting

Western blotting was performed on homogenate prepared from frozen brain samples from 5–6 mice per group. Western blots were performed on PK-digested acetone-precipitated PrP^Sc^ samples. After centrifugation pellets were re-suspended in 1X LDS loading dye (Life Technologies) + 5% β-mercaptoethanol, boiled for 5 minutes, loaded onto a 12% NuPAGE Bis-Tris gel, and run in 1x MOPS Buffer (Life Technologies). Proteins were transferred to PVDF by tank blotting at 25 V for 45 minutes. The membrane was then preblocked for 30 minutes with TBST (Tris-Buffered Saline + 0.1% Tween-20) + 3% BSA (Bovine Serum Albumin) before the addition of anti-PrP mouse monoclonal antibodies 6H4 (Prionics) at a 1:10,000 dilution either for 1 hr at room temperature (RT) or overnight (O/N) at 4 C. A secondary biotinylated sheep anti-mouse antibody and an HRP-conjugated streptavidin incubation step were performed for 40–60 minutes each, each in TBST + 3% BSA, and blots were washed 3 times for 5 minutes each time between antibody and streptavidin steps. Blots were developed with ECL Plus (Pierce) and imaged by fluorescence on a GBOX (Synoptics, Cambridge, UK).

### Antigen-capture enzyme immunoassay (EIA)

The IDEXX HerdChek BSE-Scrapie Ag EIA plate (Westbrook, ME) was used with modifications for the EIA-based fibril stability assay and the determination of PrP^Sc^ levels. The capture surface of the IDEXX EIA is a proprietary ligand that is specific for misfolded protein with detection of bound protein by a PrP-specific antibody, and does not require protease digestion to distinguish PrP^C^ from PrP^Sc^. Brain samples were prepared as 20% w/v homogenates in 1X PBS (phosphate-buffered saline, lacking calcium and magnesium) and homogenized in a beadbeater.

### Calculation of amount of PrP^Sc^ versus incubation period

The relative amount of PrP^Sc^ in donor elk inocula and donor mouse brains was determined using an EIA-based assay as described previously^5^. Briefly, 1% w/v brain homogenates were serially diluted in 1X PBS and tested using the EIA assay and diluted until the OD_450_ readings were in the linear range of detection. We have previously determined the assay range across which a dilution series of infected brain homogenate approximates a linear function, which was utilized for concentration estimation and the fibril stability assay. To provide a normalization metric across multiple samples, the 1% (w/v) homogenate was assigned a brain unit equivalent (BU) value of 100 and equivalent BU’s were calculated for each dilution, i.e. 1:2 dilution = 50 BU, 1:4 dilution = 25 BU. For each sample, the EIA OD reading in the linear range (minus the negative control value) was divided by the BU of the dilution at which the linear range OD was measured, to generate an OD/BU value. We then calculated the ratio of the OD/BU values for each sample as compared to the sample with the lowest OD/BU value. Ratio values were plotted against incubation period.

### EIA-based fibril stability assay

PrP^Sc^ fibril stability was determined using an EIA-based assay as described previously^24^. Briefly, dilutions of mouse brain samples were incubated at concentrations of guanidine hydrochloride (GdnHCl) over a range from 0.25 M to 4.0 M. Brain homogenate was diluted up to 8X into PBS in order to bring the final EIA optical density (OD_450_) in the ELISA well to 1.0 or below. A final concentration of brain homogenate of ≤ 2% (w/v) was used in all samples to avoid exceeding the buffering capacity, as was observed to occur at higher concentrations of brain homogenate^23^. The relative amount of PrP^Sc^ remaining, as assessed by the OD_450_ after dilution to 0.25 M GdnHCl, was plotted against GdnHCl concentration. For each inoculation group, the assay was applied to 4–8 mice with 3–8 technical replicates per mouse. The mean EIA OD values for each GdnHCl concentration were calculated across biological replicates and used to generate a curve. The midpoint of the curve, or [GdnHCl]_1/2_, is defined as the concentration of GdnHCl at which the PrP^Sc^ signal was reduced by half of the signal at 0.25 M GdnHCl; PrP^Sc^ with a smaller [GdnHCl]_1/2_ is less stable. As described previously^23^, due to variations in the upper baseline shape, the Smooth Line function in Microsoft Excel was used to connect data points in each curve (for each individual animal) and visualize the midpoint. In order to assess statistical differences, Welch’s ANOVA (accounting for unequal variances) was used, with the Games-Howell test for (non-parametric) post-hoc analysis. Analysis was conducted in two batches: (a) across values normalized to 0.25 M, and (b) across values normalized to 1 M.

### Recombinant prion protein production and purification

*E. coli* BL21(λDE3) was transformed with a pET28a vector containing the elk PrP gene corresponding to mature length PrP (amino acids 23–231, GenBank accession number AAC12860.2), and elk recombinant PrP was expressed and purified as described for bovine PrP^35,36^. The concentration of filtered protein eluent was determined by UV absorbance at 280 nm using an extinction coefficient of 59485 M^−1^cm^−1^ as calculated for mature length elk prion protein.

### Real-time quaking induced conversion (RT-QuIC)

Brain homogenate from one mouse from each of the first passage groups and two mice from each of the second passage groups was used as seeds for the RT-QuIC reactions. A 98 µL volume of RT-QuIC reaction mixture consisting of 10 mM phosphate buffer (pH 7.4), 300 mM NaCl, 0.1 mg/ml recombinant elk prion protein (132 L or 132 M), 10 µM thioflavin T (ThT), and 1 mM ethylenediaminetetraacetic acid tetrasodium salt (EDTA) was loaded into each well of a black 96-well plate with a clear bottom (Nunc, Thermo Fisher Scientific), and 2 µL of normalized brain homogenate dilution was added. The plate was then sealed and incubated in a BMG FLUOstar Omega plate reader at 42 °C for 100 h with cycles of 15 min shaking (700 rpm, double orbital pattern) and 15 min rest. All reactions for each sample were performed in 8 replicates. To be considered positive, the ThT fluorescence of at least two replicate reactions must be positive and above the pre-defined positive threshold that was calculated as 10 standard deviations above the mean fluorescence of normal elk brain homogenates^37–39^.

## Supplementary information

Supplementary information. 

## Data Availability

All data generated or analysed during this study are included in this published article (and its Supplementary Information files).
