# Supplementary material for: Novel Strain of the Chronic Wasting Disease Agent Isolated From Experimentally Inoculated Elk With LL132 Prion Protein
Source: Sci Rep. 2020 Feb 21;10:3148. doi: 10.1038/s41598-020-59819-1 (PMC7035384; doi:10.1038/s41598-020-59819-1)
Supplement: Supplementary file 1 — Supplementary information. [file 41598_2020_59819_MOESM1_ESM.pdf]

Novel Strain of the Chronic Wasting Disease Agent Isolated From Experimentally Inoculated Elk With LL132 Prion Protein

Jo Moore[1], Trudy Tatum[1], Soyoun Hwang[1], Catherine Vrentas[1], M. Heather West Greenlee[2], Qingzhong Kong[3], Eric Nicholson[1], and Justin Greenlee[1,*]

[1]USDA, Agricultural Research Service, National Animal Disease Center, Virus and Prion Research Unit, Ames, 50010, USA
[2]Iowa State University, Department of Biomedical Sciences, Ames, 50010, USA
[3]Case Western Reserve University, Departments of Pathology and Neurology, Cleveland, 44106, USA

[*]justin.greenlee@ars.usda.gov

Supplementary Information

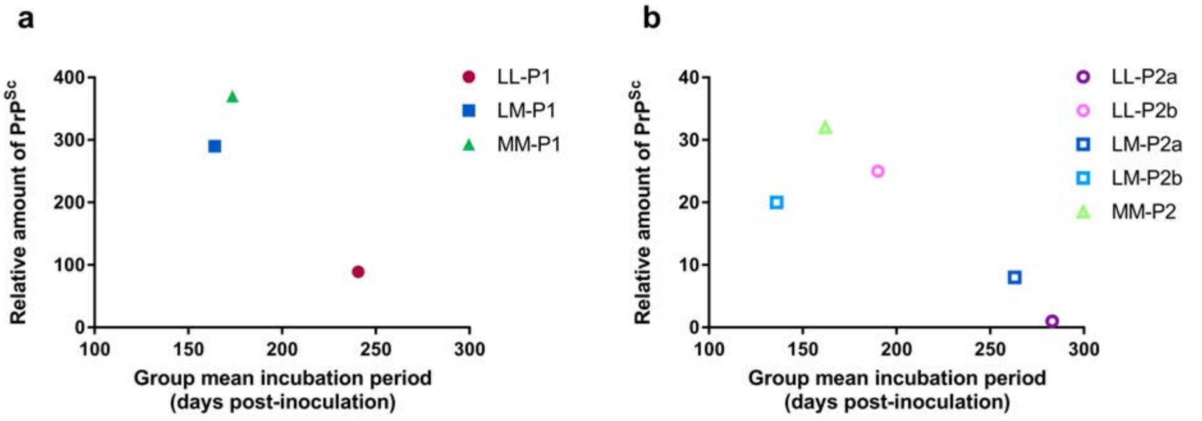

Fig S1. Group mean incubation period versus relative amount of PrP^{Sc} in the donor inoculum for first (a) and second (b) passage mice. Donor elk were used to inoculate mice in the first passage studies. Selected donor mice from the first passage studies were used to inoculate mice in the second passage studies. Note that y-axis values are 10x higher in the first passage graph (a) compared to the second passage (b). The amount of PrP^{Sc} in each inoculum was calculated using optical density readings from an antigen-capture enzyme immunoassay (EIA). The relative amount of PrP^{Sc} in the inoculum with the lowest EIA result in the linear range (LL-P2a) was designated a baseline value of 1.0. Results of other inocula are expressed as a ratio relative to the baseline inoculum. All mice express M132 elk prion protein. In mouse group names LL/LM/MM indicates the prion protein genotype of the original elk donor for the first passage study i.e. LL132, LM132 and MM132, respectively.

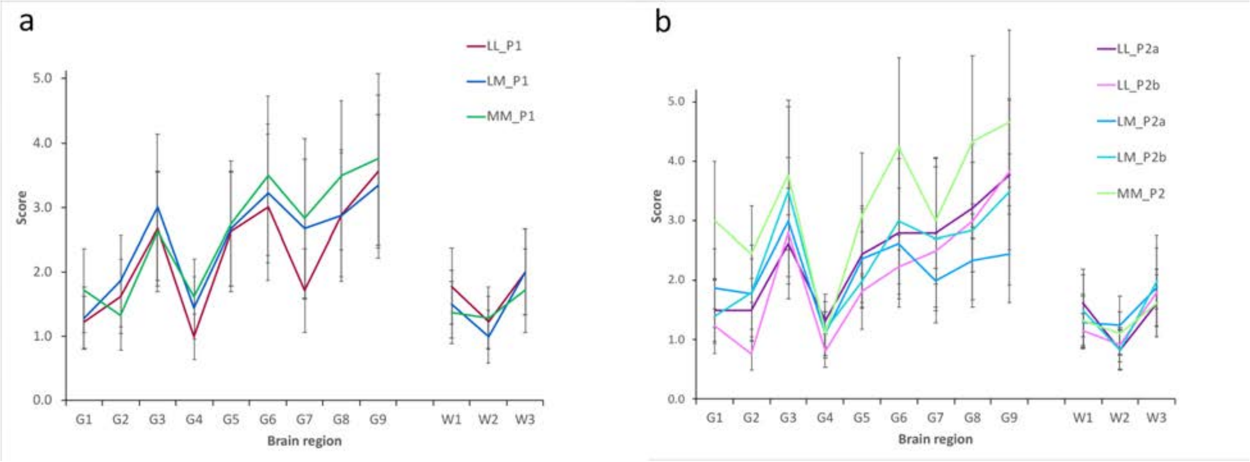

Fig S2. Vacuolation lesion profiles for first (a) and second (b) passage mice. All mice express M132 elk prion protein. In mouse group names LL/LM/MM indicates the prion protein genotype of the original elk donor for the first passage study i.e. LL132, LM132 and MM132, respectively. Error bars reflect the SEM (standard error of the mean). Neuroanatomical areas: G1, dorsal medulla nuclei; G2, cerebellar cortex of the folia including the granular layer, adjacent to the fourth ventricle; G3, cortex of the superior colliculus; G4, hypothalamus; G5, thalamus; G6, hippocampus; G7, septal nuclei of the paraterminal body; G8, cerebral cortex (at the level of G4 and G5); G9, cerebral cortex (at the level of G7); W1, cerebellar white matter; W2, midbrain white matter; W3, cerebral peduncle) [20].
